# Differentiating Between Enantiomers with Nuclear Quadrupole Coupling Using Microwave Three-Wave Mixing

**DOI:** 10.1021/acs.jpclett.5c02849

**Published:** 2025-11-11

**Authors:** Freya E. L. Berggötz, Monika Leibscher, Wenhao Sun, Christiane P. Koch, Melanie Schnell

**Affiliations:** † 28332 Deutsches Elektronen-Synchrotron DESY, Notkestr. 85, 22607 Hamburg, Germany; ‡ Institut für Experimentalphysik, Universität Hamburg, Luruper Chaussee 149, 22761 Hamburg, Germany; ¶ 9166 Freie Universität Berlin, Fachbereich Physik and Dahlem Center for Complex Quantum Systems, Arnimallee 14, 14195 Berlin, Germany; § Institut für Physikalische Chemie, Christian-Albrechts-Universität zu Kiel, Max-Eyth-Straße 1, 24118 Kiel, Germany

## Abstract

We demonstrate the application of microwave three-wave mixing to the amino alcohol valinol, which displays a hyperfine structure in the rotational spectrum due to nuclear quadrupole coupling. The hyperfine structure complicates the typical triad of rotational states, leading to overlapping microwave three-wave mixing cycles. We identified a set of cycles accessible within the hyperfine substructure of the rotational states |*J*_*K*_*a*_ *K*_*c*__⟩ = |1_01_⟩, |2_12_⟩, and |2_02_⟩ by applying the selection rules for rotational transitions and nuclear quadrupole coupling. To address an individual cycle of hyperfine transitions or subsets of cycles simultaneously, we explored different pulse schemes exploiting single-frequency or chirped microwave pulses. Each pulse scheme generated a distinct chiral signal, which shows clear enantiomer differentiation. The experimental findings agree very well with numerical simulations using an effective model for the hyperfine interaction. This study thus extends the applicability of microwave three-wave mixing to previously unexplored molecular systems containing quadrupolar nuclei.

## Graphical abstract

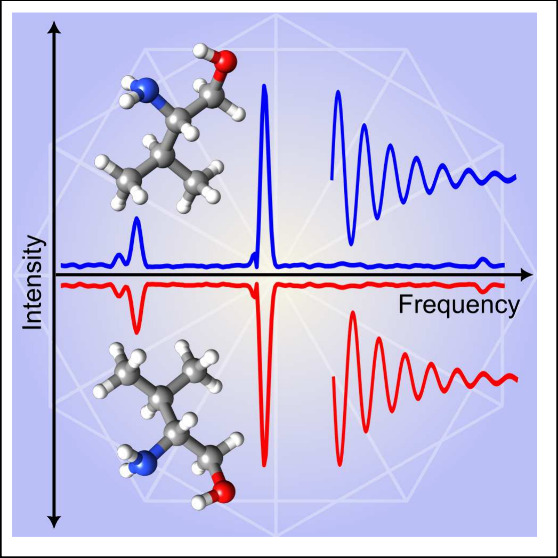

Chirality is a ubiquitous property in nature that plays an important role in biology, biochemistry, and chemistry. Molecular chirality refers to molecules that cannot be superimposed with their mirror images; they exist as right- and left-handed enantiomers. Although their physical properties, such as boiling or melting point, are identical, their chemical and biological characteristics can differ dramatically. Most biomolecules are chiral, and interestingly, there exists a bias toward their handedness: In living organisms, sugars occur predominantly as right-handed (*D*-enantiomer) and amino acids as left-handed (*L*-enantiomer).^1^ Importantly, many biologically relevant chiral molecules, such as amino acids or nucleobases, contain nitrogen (N) atoms, especially in the form of amino (NH_2_) groups.^2^

Enantiomers are recognized as distinct structures when they interact with the chiral receptors in our bodies, directly linking their biological functionality to their handedness.^3^ This has a great impact on the effectiveness and toxicity of pharmaceuticals, considering that over half of all marketed drugs today consist of chiral compounds.^4^ Thus, reliable methods are needed to distinguish molecular handedness and determine the enantiomeric excess (ee) in molecular mixtures.

Rotational spectroscopy is a powerful technique for characterizing gas-phase molecular structures, as each molecule exhibits a unique rotational fingerprint. The rotational energy levels and the corresponding transition frequencies depend on the rotational constants *A*, *B*, and *C*, which are inversely related to the moments of inertia *I*_*a*_, *I*_*b*_, and *I*_*c*_. The signal intensity scales with the square of the electric dipole-moment components, μ_*a*_, μ_*b*_, and μ_*c*_. For molecules containing quadrupolar nuclei, rotational spectra also provide insight into electronic structure. For example, a nitrogen atom, ^14^N, with a nuclear spin of *I* = 1 in a molecule induces nuclear quadrupole coupling (NQC), observable as a hyperfine splitting of the rotational transitions. The NQC constants extracted from these splittings reflect the interaction between the nuclear quadrupole moment and the local electric field gradient, thereby offering direct information about the electronic structure, e.g., the character of chemical bonds.^5–7^ Due to the high structural sensitivity of rotational spectroscopy, not only can different molecular species be distinguished, but also their isomers, diastereomers, conformers, and isotopologues. However, since the rotational constants of enantiomers are almost identical - except for a minor difference due to the parity-violating effect of the weak interaction^8^ - it is not possible to differentiate between their handedness using conventional rotational spectroscopy.

Around a decade ago, microwave three-wave mixing (M3WM) emerged as a powerful tool for chiral differentiation and quantification.^9,10^ This coherent, resonant, and nonlinear technique presents an extension of conventional rotational spectroscopy and is based on the fact that the total electric dipole moment is mirrored for the enantiomeric pair, which is reflected in a sign flip of the scalar triple product of the transition dipole moments **μ**_**a**_ · (**μ**_**b**_ × **μ**_**c**_). By driving two transitions of a closed-loop three-level system composed of all three dipole-allowed types of rotational transitions (*a*-, *b*-, and *c*-type) with two resonant microwave fields of orthogonal polarization, a chiral signal of the molecular system can be obtained in the third mutually orthogonal direction that closes the loop. The excitation of these two transitions, called the “drive” and “twist”, induces a molecular response termed the “listen” transition, which is recorded in the form of a free induction decay (FID). The phase of the measured signal differs by π radians for the two different enantiomers. The signal is directly proportional to the ee of the sample and can therefore be used to quantitatively determine the ee.^11^ The listen signal, *P*_*listen*_, with the listen frequency ν_*L*_ can be expressed in a simplified form as

$$P_{\mathrm{listen}}\left(t\right)\propto ee\cdot \left|\mu_{a}\mu_{b}\mu_{c}\right|\cdot \cos\left(2\pi \nu_{L}t+\frac{\pi }{2}\frac{\mu_{a}\mu_{b}\mu_{c}}{\left|\mu_{a}\mu_{b}\mu_{c}\right|}\right)$$ 1

where μ_*i*_ (*i* = *a*, *b*, *c*) are the dipole moment components along the *a*, *b*, and *c* principal inertia axes.^12^ For enantiopure samples, the magnitude of the signal is on the order of conventional chirped-pulse Fourier transform microwave (CP-FTMW) spectroscopy signals. To perform M3WM spectroscopy on a molecular system, the molecules must be brought into the gas phase and all three dipole moment components μ_*a*_, μ_*b*_, and μ_*c*_ must be nonzero. That is, the molecular system must belong to the *C*_1_ point group.

M3WM was first experimentally demonstrated with the chiral molecule 1,2-propanediol using a cryogenic helium buffer gas cell for cooling (*T*_rot_ ∼ 7 K).^10^ Shortly after, the technique was successfully applied to mixtures of chiral molecules cooled in a supersonic jet.^13^ Various chiral molecular systems have been investigated using not only sequential single-frequency pulse schemes but also time-overlapping as well as chirped drive and twist pulses.^14,15^ In addition to determining the ee and differentiating the enantiomers of chiral molecules, advanced M3WM pulse schemes have enabled enantiomer-selective population transfer and the controlled separation of the enantiomeric pair into different specified rotational states.^16–20^ The underlying population dynamics of the rotational states involved in M3WM and its extension to enantiomer-selective population transfer have inspired different theoretical studies such as the determination of optimal excitation schemes using coherent control^21,22^ or the influence of spatial degeneracy.^23,24^

Here, we demonstrate the application of M3WM to a nitrogen-containing molecule, namely valinol, which has been studied with CP-FTMW spectroscopy before,^25^ extending the applicability of M3WM to this relevant class of molecules. Due to NQC in general, and the particularly narrow hyperfine split patterns of ^14^N nuclei, new challenges arise, i.e., overlapping M3WM cycles and off-resonant excitations. With a combined experimental and theoretical approach, we investigate the possibility of enantiomer differentiation in such systems and discuss different M3WM schemes for selective and combined excitation of the overlapping M3WM cycles.

The experimental setup (Figure 1a) is based on the CP-FTMW spectrometer COMPACT, which was modified to perform M3WM measurements, as reported elsewhere.^26,27^ *L*- and *D*-valinol were purchased from Santa Cruz Biotechnology and Sigma-Aldrich, with a stated chemical purity of 99.9% and 98.5%, respectively, and used without further purification. The samples were placed in a reservoir and heated to around 36 °C, which is slightly above the melting point. The reservoir is connected to a pulsed nozzle (modified General valve Series 9), operating at a repetition rate of 6 Hz. Using helium as the carrier gas at a stagnation pressure of around 2.5 bar, the sample was supersonically expanded into the vacuum chamber, leading to a rotational temperature of the molecules of approximately 2K.

**Figure 1:**
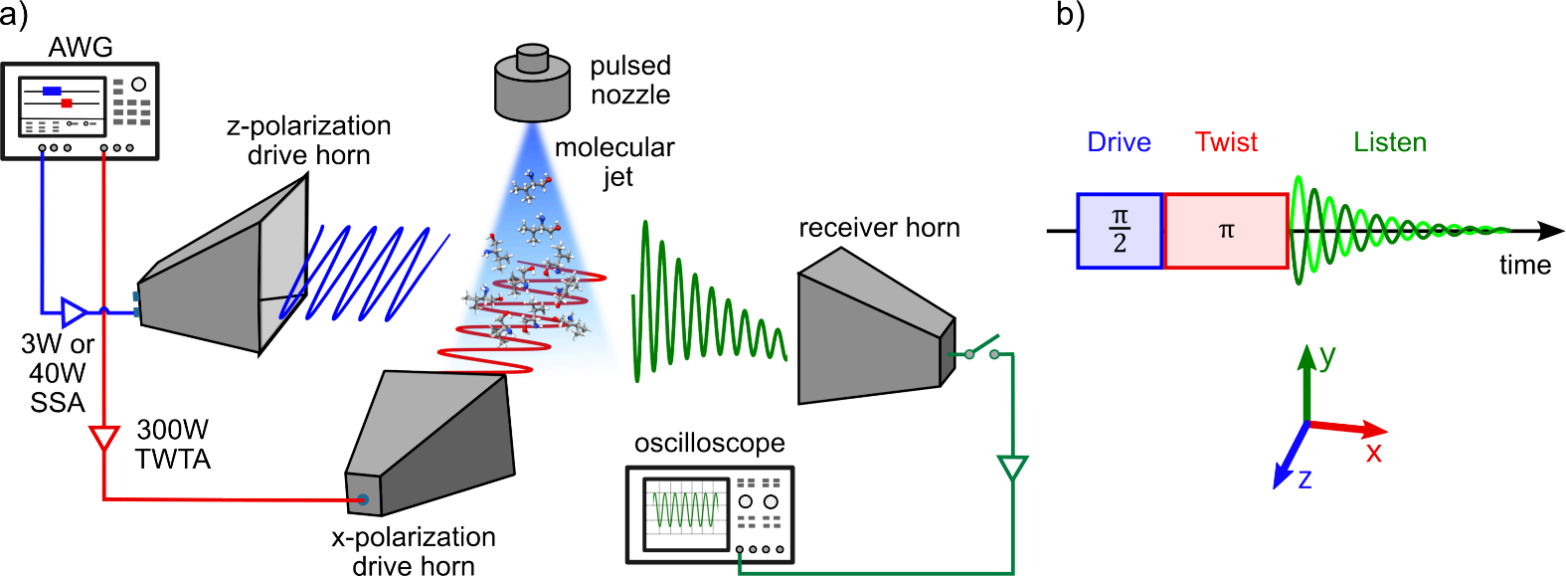
(a) Scheme of the experimental setup: The two excitation pulses are generated by an arbitrary waveform generator (AWG). Two horn antennas broadcast the microwave pulses in two different polarization directions into the vacuum chamber, where they sequentially interact with the supersonically cooled molecular jet. The molecular response is detected in the third polarization direction by a horn antenna and digitized by an oscilloscope. (b) Pulse scheme of the microwave three-wave mixing experiment in the time domain. The coordinate system represents the polarization directions of the excitation pulses (*z*, *x*) and the signal, *P*_listen_ (*y*).

Two horn antennas broadcast the microwave pulses generated by a two-channel arbitrary waveform generator (AWG) in orthogonal polarization directions (*z* and *x*) into the vacuum chamber. Different amplifiers were used for the two pathways, that is, a 3W or a 40W solid state amplifier (SSA) for the drive pulse and a 300W traveling-wave tube amplifier (TWTA) with adjustable gain for the twist pulse. Inside the vacuum chamber, the microwave pulses sequentially interact without any delay between the pulses with the molecular ensemble, generating the listen signal in the third mutually orthogonal polarization direction (*y*), see Figure 1b. This signal was detected by a receiving horn antenna on the detection side in the form of a free induction decay. Using the fast-frame option of the oscilloscope, six M3WM experiments were performed for each gas pulse, leading to an effective repetition rate of 36 Hz.

For an asymmetric rotor molecule, every rotational energy level is labeled as |*J*_*K*_*a*_ *K*_*c*__⟩ according to the King-Hainer-Cross notation where the quantum number *J* represents the total angular momentum and the pseudoquantum numbers *K*_*a*_ and *K*_*c*_ denote the projection on the principal *a*- and *c*-axes, respectively. Each rotational energy level is further (2*J* + 1)-fold degenerate with respect to the orientational quantum number *M*_*J*_. The selection rule for dipole-allowed transitions is Δ*J* = +1, 0, –1, giving rise to the *R*-, *Q*-, and *P*-branch in the rotational spectrum, respectively. Due to this selection rule, a closed cycle of dipole-allowed rotational transitions necessary for M3WM is only possible if ∑_*i*_Δ*J*_*i*_ = 0, with *i* referring to the drive, twist, and listen transitions. Thus, the cycle consists purely of *Q*-branch transitions or a combination of *R*-, *P*-, and *Q*-branches transitions.^23^

Molecules containing an atom with nuclear spin *I* > 1/2 exhibit a hyperfine structure of their rotational transitions due to NQC. The nuclear spin **I** and the angular momentum **J** couple, resulting in the total angular momentum **F** = **J** + **I**, which is characterized by the quantum number *F* = *J* + *I*, *J* + *I* – 1, ..., |*J* – *I*|. Each state with the quantum number *F* is (2*F* + 1)-fold degenerate in *M*_*F*_, with *M*_*F*_ ranging from –*F* to *F*.

Due to the quadrupole hyperfine structure introduced by the ^14^N atom, the typical three-level system used in M3WM expands into a more complex nine-level system. Figure 2 illustrates the relevant rotational energy levels of valinol—|1_01_ *F*⟩, |2_12_ *F′*⟩, and |2_02_ *F*″⟩—including their hyperfine structure and all dipole-allowed transitions, revealing several overlapping M3WM cycles that can be addressed experimentally. By employing different microwave pulse schemes, we aim to selectively excite either individual cycles or sets of cycles within this manifold and demonstrate that an enantiomer selective signal can be measured in all cases, despite the complexity of the level structure due to the hyperfine splitting. The experimental results are compared to numerical simulations of the corresponding time-dependent Schrödinger equation using an effective model based on the nine-level system shown in Figure 2.

**Figure 2:**
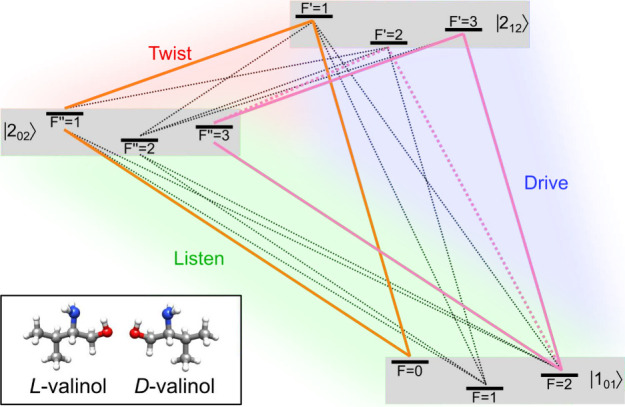
Energy-level scheme for the M3WM cycle for valinol including the hyperfine splitting caused by the ^14^N NQC: All drive |*J*_*K*_*a*_ *K*_*c*__ *F*⟩ → |*J*_*K*_*a*_^*′*^ *K*_*c*_^*′*^_^′^ *F′*⟩ = |1_01_ *F*⟩ → |2_12_ *F′*⟩ (*b*-type), twist |2_12_ *F′*⟩ → |2_02_ *F*″⟩ (*c*-type), and listen transitions |2_02_ *F*″⟩ → |1_01_ *F*⟩ (*a*-type) are depicted. The black dotted lines show all the dipole-allowed transitions, while the closed cycles leading to the *F*″ → *F* = 3 → 2 and the *F*″ → *F* = 1 → 0 listen signal are highlighted in pink and in orange, respectively. The degeneracy with respect to the orientational quantum number *M*_*F*_ was omitted for the sake of clarity. The magnitudes of the dipole moment components |μ_*a*_|, |μ_*b*_|, and |μ_*c*_| for the lowest energy conformer are 3.0, 1.2, and 0.7 D, respectively, as obtained at the B3LYP-D3­(BJ)/aug-cc-pVTZ level of theory.^25^

An overview of all drive transitions |1_01_ *F*⟩ → |2_12_ *F′*⟩ with connecting twist (|2_12_ *F′*⟩ → |2_02_ *F*″⟩) and listen (|2_02_ *F*″⟩ → |1_01_ *F*⟩) transitions forming a closed loop is given in Table 1. For certain combinations of states and the quantum number *F*, which obeys the selection rule Δ*F* = 0, ±1, none of the listen transitions can complete the M3WM cycle. As summarized in Table 1, a closed transition cycle can only be found if Δ*F* obeys the same condition as Δ*J* for M3WM, that is, ∑_*i*_Δ*F*_*i*_ = 0 where *i* represents drive, twist, and listen. Thus, possible combinations of Δ*F* are 0, 0, 0 or any permutation of 0, +1, −1. Therefore, up to three closed M3WM cycles per listen transition can exist (i.e., for listen transitions with Δ*F* = 0, since otherwise the first combination option is not possible). A total of 12 closed M3WM cycles are possible for the present level scheme (Table 1).

**Table 1:** Overview of the Different Hyperfine Transitions for the Drive, Twist, and Listen Transitions^a^

Drive: |1_01_⟩ → |2_12_⟩ Frequency/MHz (*F* → *F′*)^b^	Twist: |2_12_⟩ → |2_02_⟩ Frequency/MHz (*F′*→ *F*″)^c^	Listen: |2_02_⟩ → |1_01_⟩ Frequency/MHz (*F*″ → *F*)^d^
7874.51 (0 → 1)	1955.82 (1 → 1)	5918.69 (1 → 0)
	1957.86 (1 → 2)	-
7875.52 (2 → 2)	1954.95 (2 → 1)	5920.57 (1 → 2)
	1955.68 (2 → 3)	5919.84 (3 → 2)
	1956.99 (2 → 2)	5918.53 (2 → 2)
7876.08 (2 → 3)	1956.24 (3 → 3)	5919.84 (3 → 2)
	1957.55 (3 → 2)	5918.53 (2 → 2)
7876.39 (2 → 1)	1955.82 (1 → 1)	5920.57 (1 → 2)
	1957.86 (1 → 2)	5918.53 (2 → 2)
7876.77 (1 → 2)	1954.95 (2 → 1)	5921.82 (1 → 1)
	1955.68 (2 → 3)	-
	1956.99 (2 → 2)	5919.78 (2 → 1)
7877.64 (1 → 1)	1955.82 (1 → 1)	5921.82 (1 → 1)
	1957.86 (1 → 2)	5919.78 (2 → 1)

a The transition frequencies are based on the fitted rotational constants determined by Arenas et al.^25^

b Six hyperfine components of the drive transition (|1_01_ *F*⟩ → |2_12_ *F′*⟩).

c Twist transitions (|2_12_ *F′*⟩ → |2_02_ *F*″⟩) that connect to the respective drive transition.

d Listen transitions (|2_12_ *F*″⟩ → |2_02_ *F*⟩), which connect with the initial state and form a closed loop of transitions.

To investigate enantiomer differentiation in the presence of hyperfine structure, three microwave pulse schemes were tested: two based on single-frequency (SF) excitation and one using chirped pulses. The schemes target either an individual M3WM cycle or the entire set of possible cycles within the selected energy level system (Figure 2). Each approach is evaluated for its ability to distinguish enantiomers. In addition, the selectivity of the SF sequences is analyzed, and the accuracy of the effective nine-level model is assessed by comparing the simulations of the underlying population dynamics in the rotational states with the experimental data for each pulse scheme.

For scheme I, SF microwave pulses excite the drive transition |*J*_*K*_*a*_ *K*_*c*__ *F*⟩ → |*J*_*K*_*a*_^*′*^ *K*_*c*_^*′*^_^*′*^ *F′*⟩ = |1_01_0⟩ → |2_12_1⟩ at 7874.51 MHz and the twist transition |2_12_1⟩ → |2_02_1⟩ at 1955.82 MHz, see Table 2. The listen transition |2_02_1⟩ → |1_01_0⟩ closes the loop (Table 1). As highlighted in Figure 2 in orange and apparent from Table 1, no other M3WM cycle can generate this listen signal. Thus, the triad addressed by pulse scheme I resembles an isolated three-level system used in M3WM spectroscopy in molecules without hyperfine structure.

**Table 2:** Summary of the Three Different Pulse Schemes with Excitation Frequencies and Experimentally Determined Pulse Durations for the Drive and the Twist Pulse, Respectively

	Drive pulse		Twist pulse	
Pulse scheme	Frequency (MHz)	Duration (μs)	Frequency (MHz)	Duration (μs)
I^a^	7874.51	1.2	1955.82	2.6
II^a^	7876.08	1.4	1956.24	2.4
III^b^	7876.08 ± 5	0.3	1956.41 ± 5	0.2

a Amplifier for drive pulse: 3 W SSA, amplifier for twist pulse: 300 W TWTA with 10% gain.

b Amplifier for drive pulse: 40 W SSA, amplifier for twist pulse: 300 W TWTA with 65% gain.

Scheme II, highlighted with pink solid lines in Figure 2, combines an SF drive and twist pulse exciting the transitions |1_01_2⟩ → |2_12_3⟩ at 7876.08 MHz and |2_12_3⟩ → |2_02_3⟩ at 1956.24 MHz, respectively, resulting in the listen signal at a transition frequency of 5919.84 MHz (Table 1). Another cycle overlaps with the listen transition, namely the |1_01_2⟩ → |2_12_2⟩ → |2_02_3⟩ cycle, highlighted with dotted pink lines in Figure 2. Here, the hyperfine structure causes overlapping cycles that could affect the ability to distinguish enantiomers.

Finally, scheme III employs a chirped drive and twist pulse covering the frequency range of the entire hyperfine structure of the respective transition, see Table 2. The frequencies are linearly swept across the transition’s center frequency (defined as the midpoint between the lowest and highest hyperfine components, i.e., 7876.08 and 1956.41 MHz for drive and twist transitions, respectively) with a bandwidth of Δω = 10 MHz, which is sufficient to cover the hyperfine structure of each transition. The three excitation schemes were expected to generate distinct listen signals according to Table 1. For each scheme, the optimized pulse durations and the amplifiers used are summarized in Table 2.

The durations of the drive and twist pulses, τ_*d*_ and τ_*t*_, were optimized by recording nutation curves, that is, measuring the transition intensity as a function of the pulse duration. Carefully selecting the amplification power and pulse duration can enhance the signal of the desired transition and reduce the coexcitation of nearby transitions. First, the drive pulse polarized in *z* direction generates a coherent superposition between the states |1_01_ *F*⟩ and |2_12_ *F′*⟩. For the resonant SF drive pulses with electric field strength *E*, the maximal signal in the nutation curve corresponds to a Rabi flip angle Θ = Ωτ_*d*_ = π/2, where $\Omega =\frac{\mu E}{\hbar }$ is the Rabi frequency, i.e., to maximum coherence and a 50/50 population distribution between the two states. Note that the Rabi flip angles vary with the quantum number *M*_*F*_, thus an effective π/2-condition averaging over all *M*_*F*_ substates is experimentally determined.^28^ For nonresonant excitation or excitation with a chirped pulse, the maximal signal intensity does not necessarily refer to a 50/50 coherence. Here, a comparison with numerical simulations of the population dynamics is used to evaluate the population transfer, which is discussed in more detail below.

The twist pulse is optimized for the π condition, however, the intensity of the respective transition cannot be measured directly since there is no detection horn antenna in the *x*-polarization direction (Figure 1). Instead, the M3WM signal was recorded using the optimal drive pulses, and the duration of the twist pulse was optimized for maximum listen signal.

For a deeper understanding of the underlying population dynamics in the different rotational states, the nutation curves were numerically simulated using the effective nine-level model depicted in Figure 2, where the *M*_*F*_-degeneracy of each state is accounted for by averaging over the degenerate states. Details of the numerical simulations and a comparison of the measured and simulated nutation curves are given in the Supporting Information (Figures S2, S3, and S4). The very good qualitative and quantitative agreement of the experimental and simulated nutation curves allows us to draw conclusions on the excitation process from the simulations employing the effective nine-level model.

Using the optimized pulse durations, the M3WM signal was measured for *L*- and *D*-valinol for each pulse scheme. The resulting spectra along with the simulated signal are depicted in Figure 3. As the signals were not calibrated, the listen signal of *D*-valinol appears slightly weaker compared to that of *L*-valinol. Analyzing the phases of the individual hyperfine transitions demonstrates clear enantiomer differentiation: The phase of the complex Fourier transformed signal at the respective frequency is roughly shifted by 180° for all three schemes when the handedness of the enantiomer is changed (Table 3). For scheme III, only the phase of the strongest listen transition is given in Table 3, yet each listen frequency individually shows a phase shift by approximately 180° (Table S1 in the Supporting Information). Thus, even if multiple closed M3WM cycles are excited that can overlap and interfere with each other, an overall phase shift of 180° is obtained and enantiomer differentiation is possible. Note that the absolute phases in these experiments carry no meaningful information and that the values for the different schemes cannot be compared with each other due to the frequency-dependent dispersion of the different electronic components. A thorough calibration of the system would enable the determination of the absolute configuration, as discussed by Shubert et al.^11^

**Figure 3:**
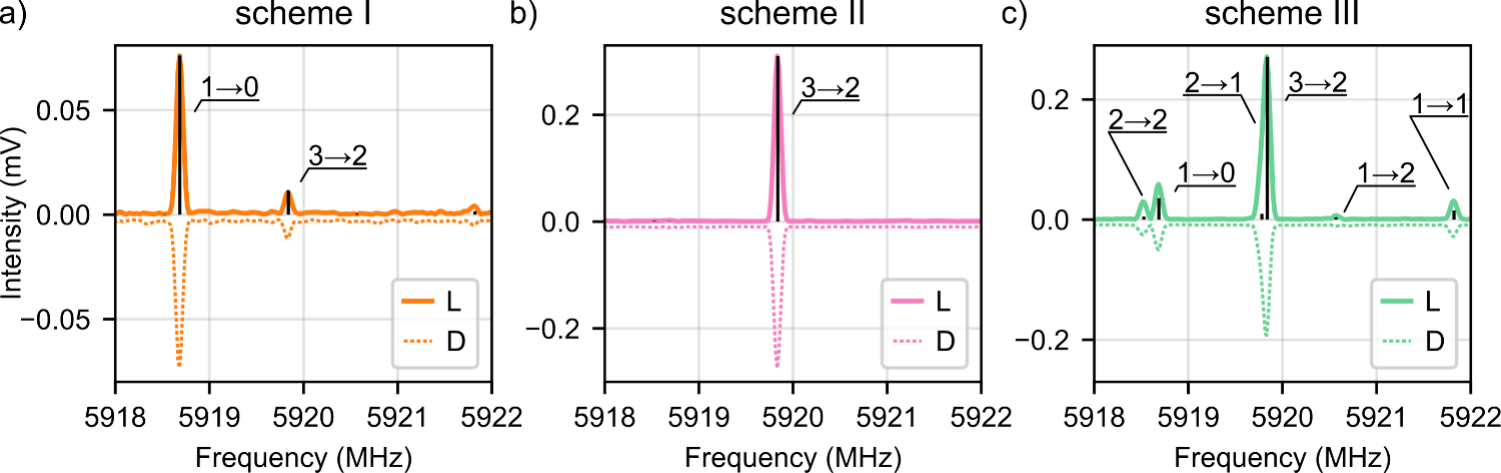
Experimental M3WM signal (10^5^ averaged FIDs) for the listen transition |2_02_⟩ → |1_01_⟩ for *L*- and *D*-valinol as solid and dashed lines. The different hyperfine components are labeled according to *F*″ → *F*. The signal of *D*-valinol was multiplied by −1 for better comparison and given a small offset to enhance the visibility of the baseline. (a) and (b) depict the listen signal of the SF pulse schemes I (orange) and II (pink). (c) shows the listen signal obtained with the chirped pulse scheme III (mint). The simulated M3WM signal for each scheme, scaled to the strongest transition, is shown in black.

**Table 3:** Absolute Phases^a^ *ϕ*_*L*_ and *ϕ*_*D*_ of Listen Transitions for *L*- and *D*-Valinol and the Relative Phase Δ*ϕ* = *ϕ*_*D*_ – *ϕ*_*L*_

Pulse scheme	ϕ_*L*_ (deg)	ϕ_*D*_ (deg)	Δϕ (deg)
I^b^	–13	178	191
II^c^	–71	110	181
III^c^	–24	154	178

a The typical uncertainty in the listen signal phase is around 10°, as shown by Shubert et al.^13^

b Phase of the transition *F*″ → *F* = 1 → 0.

c Phase of the transition 3 → 2.

Further, variations in listen-transition intensities for the different pulse schemes highlight the selective excitation of specific M3WM cycles within the hyperfine structure. Scheme I resonantly excites the cycle marked in orange in Figure 2, leading to a strong listen signal at a transition frequency of 5918.69 MHz (*F*″ → *F* = 1 → 0, Table 1) as shown in Figure 3a. An additional chiral signal is observed at 5919.84 MHz for the *F*″ → *F* = 3 → 2 transition, which may result from two overlapping M3WM cycles: *F* → *F′* → *F*″ = 2 → 3 → 3 and 2 → 2 → 3, according to Table 1. Because the hyperfine structure spans only approximately 3 MHz, the detuning of nearby transition frequencies from the excitation frequency is small. This enables off-resonant excitation, such as for the drive transitions |1_01_2⟩ → |2_12_3⟩ at 7876.08 MHz and |1_01_2⟩ → |2_12_2⟩ at 7875.52 MHz, with theoretical fractional intensities of 47% and 8%, respectively, compared to 11% for the on-resonant transition, as tabulated in Townes and Schawlow.^29^ The optimized duration of the drive pulse (τ_*d*,*I*_ = 1.2 μs) reduces excitation of the stronger 2 → 3 → 3 cycle (Figure S2a and c of the Supporting Information), but partial excitation of the 2 → 2 → 3 cycle remains, giving rise to the additional signal observed.

The MW pulse scheme II generates a listen signal with a single peak corresponding to the *F*″ → *F* = 3 → 2 transition as can be seen in the measured and simulated spectrum in Figure 3b. This signal results from the excitation of the resonant *F* → *F′*→ *F*″ = 2 → 3 → 3 cycle (solid pink lines in Figure 2). The aforementioned overlapping cycle 2 → 2 → 3 indicated by the dotted pink lines does not contribute to the signal, since the *F* → *F′* = 2 → 2 transition is off-resonant and its fractional intensity (8%) is small compared to the *F* → *F′* = 2 → 3 transition (47%).

Finally, scheme III uses sequential chirped pulses exciting all drive and, subsequently, all twist transitions, yielding signal from all possible listen transitions (Figure 3c). The excitation process can be revealed by simulating the underlying population dynamics. The three panels of Figure 4 show the population of the hyperfine states during excitation with the drive and twist pulse. We assume that initially the three lowest states, namely |1_01_0⟩, |1_01_1⟩, and |1_01_2⟩, are populated according to their degeneracy.

**Figure 4:**
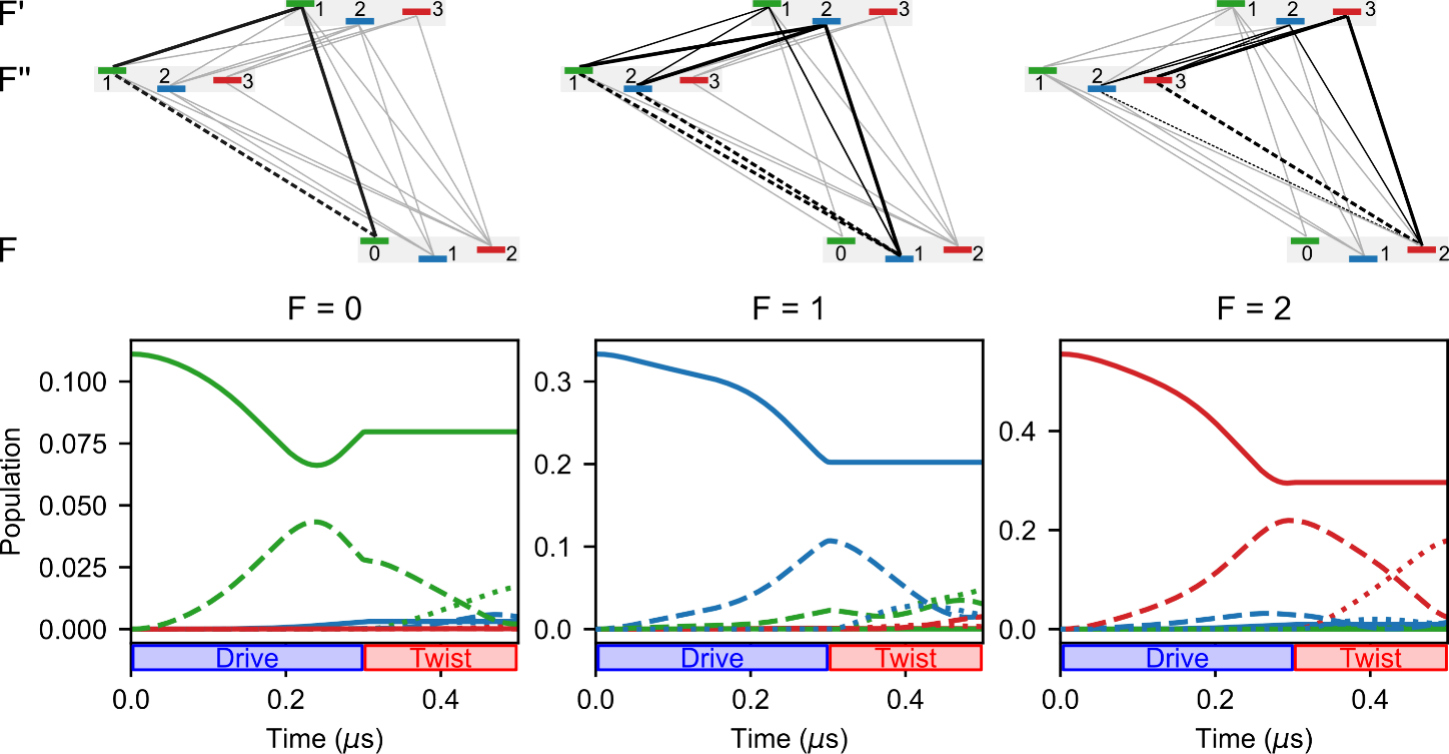
Population dynamics for the chirped scheme III, starting from the different *F* states of the |1_01_⟩ energy level. Solid, dashed, and dotted lines depict the population in the |1_01_⟩, |2_12_⟩, and |2_02_⟩ states while the different colors refer to the *F* quantum number as indicated by the level scheme on top. The initial population corresponds to the degeneracy of the rotational levels and is *P*(*F* = 0) = 1/9, *P*(*F* = 1) = 3/9, and *P*(*F* = 2) = 5/9. The level schemes further highlight the transitions that are predominantly involved in the population transfer process starting from the respective ground state over the course of the excitation.

While the 5-fold degenerate *F* = 2 state (right panel) dominates the population dynamics, the chirped pulses drive M3WM cycles (black lines in upper schemes in Figure 4) from each of the three initial states, which contribute to the listen signal. The red lines in the right panel indicate the dominant cycle, which starts from *F* = 2, resulting in the main peak of the listen signal, *F*″ → *F* = 3 → 2. Concurring cycles, cf. Table 1, contribute to the signal, especially the cycle 2 → 2 → 2 (solid red, dashed blue, and dotted blue lines). This cycle produces the listen signal *F*″ → *F* = 2 → 2, which is clearly visible in the experimental spectrum but underestimated in the simulation (see Figure 3c and right panel of Figure 4). The discrepancy arises because the electric field strength in the simulation can only be estimated, leading to larger deviations, particularly for weaker peaks.

From the initial state *F* = 1, two pairs of overlapping cycles are driven resulting in the listen signal at the transitions *F*″ → *F* = 1 → 1 and 2 → 1 (compare Table 1). The corresponding population dynamics (middle panel) shows that the 1 → 2 → 1 cycle (solid blue, dashed blue and dotted green lines), resulting in the 1 → 1 listen signal in Figure 3c, is the stronger cycle.

The population dynamics starting from the *F* = 0 level are depicted in the left panel. Here, a single M3WM cycle (indicated by the black lines in the level scheme above) governs the excitation process giving rise to the *F*″ → *F* = 1 → 0 signal shown in Figure 3. The population dynamics also reveal that the multitude of allowed transitions leads to a leak of population to states that are not part of a closed cycle and, therefore, do not contribute to the listen signal. This is particularly evident for the *F* = 1 initial state, as shown by the dashed blue, green, and red lines representing the populations of states in the 2_12_ manifold at the end of the twist pulse (middle panel, Figure 4).

For scheme III, simulating the pulse optimization was more advanced compared to schemes I and II, which were discussed above. Figure 5a and 5b show the experimental and simulated nutation curves of the drive pulse, respectively, which are in good agreement with each other. The nutation curves of the two strongest transitions, *F* → *F′* = 2 → 3 and 1 → 2, are characterized by a sharp increase followed by a slow decay of the intensity for longer pulse durations overlapped by some fast oscillation. This behavior indicates that the chirped pulse does not meet the conditions for completely adiabatic population transfer.

**Figure 5:**
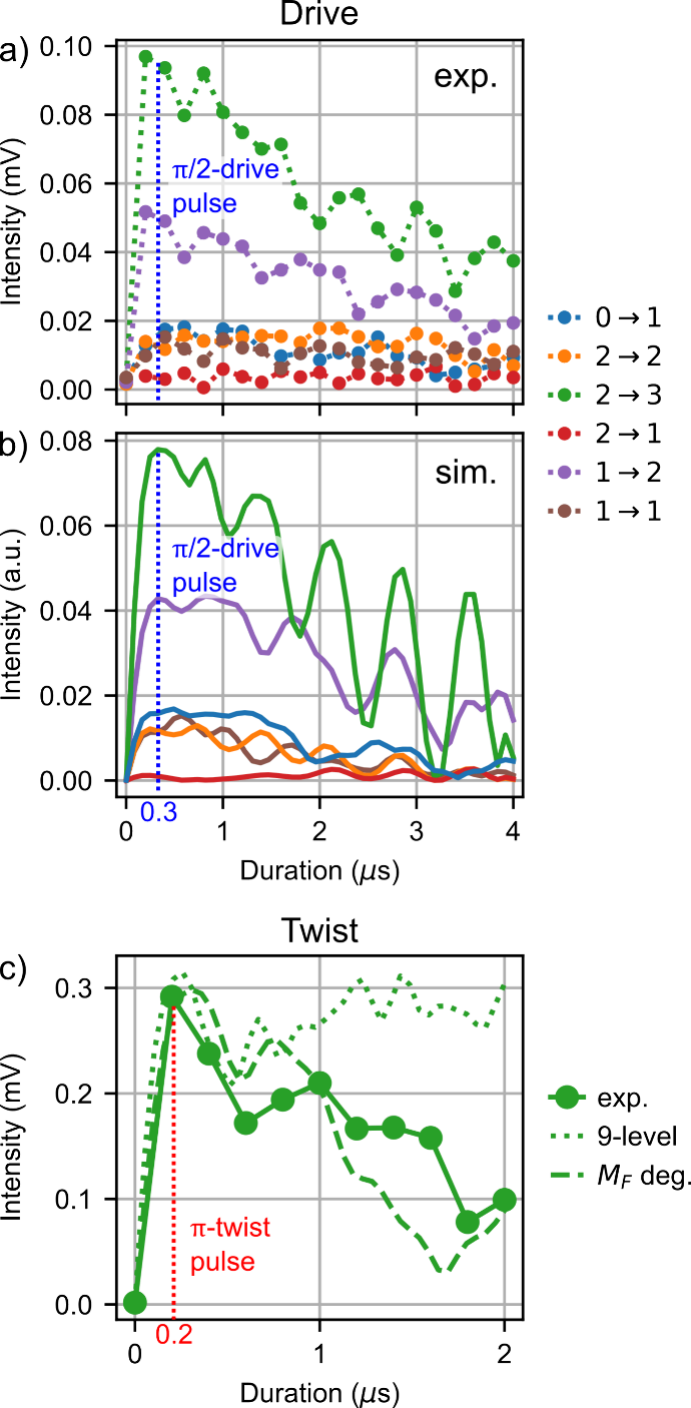
(a) and (b) depict experimental and simulated nutation curves, including all hyperfine transitions for the chirped drive of scheme III. The duration of the drive pulse was scanned from 0 to 4 μs in steps of 0.2 μs, averaging 10^4^ FIDs per time step. A shorter scan (0–2 μs) with finer step size (0.1 μs) can be found in the . (c) Comparison of the measured twist nutation curve of the transition *F*″ → *F* = 3 → 2 (scanned from 0 to 2μs in steps of 0.2 μs, averaging 10^4^ FIDs per time step) of the chirped twist pulse (solid line) to two different models (dotted and dashed lines). Only the intensity of the strongest listen transition is shown as a function of the twist pulse duration.

Due to the differing Rabi frequencies of each individual transition addressed with the chirped pulse, the pulse duration cannot be optimized to create total coherence for all transitions simultaneously. Here, simulating the corresponding population dynamics—shown in Figure 4—reveals that 50/50 coherence is approximately obtained only for the *F* → *F′* = 2 → 3 transition (red lines in right panel of Figure 4), whose contribution dominates the nutation curve. Other transitions show stronger deviation from the 50/50 coherence, cf left and middle panel of Figure 4.

Comparing the experimental and simulated twist nutation curves for the strongest hyperfine component of the listen transition, *F*″ → *F* = 3 → 2, highlights limitations of the nine-level model (Figure 5c). The measured data shows a sharp peak in intensity at short pulse durations followed by a gradual decrease with increasing τ_*t*_. The simulation reproduces the initial rise but predicts a plateau (dotted line), whereas a simulation including the degenerate *M*_*F*_ states (dashed line; details in Supporting Information) accurately captures the observed signal decrease. This is due to the selection rule Δ*M*_*F*_ = ±1 for *x*- and *y*-polarized light, which causes population transfer to adjacent *M*_*F*_ states at longer τ_*t*_ (Figure S1 in the Supporting Information). This population is lost for the M3WM signal, as the cycle does not close anymore. Still, for short pulse durations this process is negligible, and Figure 5c shows that the nine-level model is valid for pulse durations shorter than 0.5 μs, i.e., for all cases considered here. However, simulations including the degenerate *M*_*F*_ states can become relevant for a denser hyperfine structure and in particular for transitions between states with larger *F* and thus with more *M*_*F*_-states.

Overall, we have shown that a clear M3WM signal can be obtained from molecules with hyperfine structure due to NQC, which can be used for enantiomer differentiation. Three different pulse schemes exploiting single-frequency and chirped pulses were designed to address individual cycles and the entire set of possible M3WM cycles within the selected rotational level scheme, which resulted in distinct listen signals. Examining the phases of the listen signal shows that the enantiomeric pair can be differentiated even if multiple M3WM cycles within the hyperfine structure contribute to this signal. Both pulse types—single frequency and chirped—have their advantages, and the choice in future studies will depend on the specific goal. While the different pulse schemes can produce similarly strong signals and are comparable in experimental complexity, chirped pulses may be particularly beneficial for dense hyperfine structures (e.g., from multiple quadrupolar nuclei), where addressing individual transitions is impractical. Conversely, SF M3WM schemes could serve as a useful starting point for precision experiments requiring preparation of the molecular ensemble in a defined *F* state.

Comparison of the experimental data with simulation results using an effective model has revealed the origin of the different listen signals. While the effective model was found to sufficiently describe the population dynamics of the involved rotational states in a NQC molecule for short pulse durations, as validated by comparing measured and simulated nutation curves, the full *M*_*F*_-degeneracy will need to be considered for longer pulse durations and stronger intensities. Since the effect of orientational degeneracy becomes more pronounced for larger angular momenta,^28^ an extension of the model taking the full *M*_*F*_-degeneracy into account might become important, e.g., for molecules containing multiple quadrupolar nuclei.

The experiments showcase the robustness of enantiomer differentiation using M3WM and how it can be applied to an important class of chiral molecules. In the future, it will be interesting to extend M3WM to more systems exhibiting NQC, including molecular species with stronger coupling constants, such as chlorine- or even bromine- and iodine-containing molecules. Chiral molecules containing heavy atoms, which often exhibit NQC, are of high interest for detecting parity violation in molecules.^30–32^ Furthermore, using the extension of M3WM—enantiomer-selective population transfer—could enable the preparation of a chiral molecule in a designated hyperfine state in an enantiomer-selective fashion. Such a prepared and controlled sample could be the starting point for future advanced precision experiments.

## Supplementary Material





## Notes

Details on numerical simulations, nutation curves and experimental details on pulse optimization, phases of the listen signal of scheme III (PDF)
Transparent Peer Review report available (PDF)
