# Supplementary material for: Differentiating Between Enantiomers with Nuclear Quadrupole Coupling Using Microwave Three-Wave Mixing
Source: J Phys Chem Lett. 2025 Nov 11;16(46):12087–94. doi: 10.1021/acs.jpclett.5c02849 (PMC12641466; doi:10.1021/acs.jpclett.5c02849)
Supplement: Supplementary file 1 [file jz5c02849_si_001.pdf]

Supporting information:

Differentiating Between Enantiomers with Nuclear Quadrupole Coupling Using Microwave Three-Wave Mixing

Freya E. L. Berggötz,[†,‡] Monika Leibscher,[¶] Wenhao Sun,[†] Christiane P. Koch,[¶] and Melanie Schnell[*,†,§]

†*Deutsches Elektronen-Synchrotron DESY, Notkestr. 85, 22607 Hamburg, Germany*

‡*Institut für Experimentalphysik, Universität Hamburg, Luruper Chaussee 149, 22761 Hamburg, Germany*

¶*Freie Universität Berlin, Fachbereich Physik and Dahlem Center for Complex Quantum Systems, Arnimallee 14, 14195 Berlin*

§*Institut für Physikalische Chemie, Christian-Albrechts-Universität zu Kiel, Max-Eyth-Straße 1, 24118 Kiel, Germany*

E-mail: melanie.schnell@desy.de

Contents

1 Supplementary information for numerical simulation **3**

1.1 Effective 9-level model for M3WM in Valinol 3

1.2 Orientational degeneracy of the hyperfine states 5

2 Supplementary experimental details **7**

2.1 Pulse optimization and nutation curves 7

2.2 Phases of listen signal for scheme III 12

1 Supplementary information for numerical simulation

1.1 Effective 9-level model for M3WM in Valinol

We simulate the microwave three-wave mixing (M3WM) process for the hyperfine states of valinol by numerically solving the time-dependent Schrödinger equation

$$i\frac{\partial}{\partial t}|\psi(t)\rangle = (H_0 + H_{int}(t))|\psi(t)\rangle\,, \tag{1}$$

where $H_{int}(t) = -\boldsymbol{\mu}\cdot\mathbf{E}(t)$ is the interaction between the electric dipole moment $\boldsymbol{\mu}$ of the molecule and the electric field

$$\mathbf{E}(t) = \sum_{i=1,2}\mathbf{e}_i\mathcal{E}_i(t)\cos\left(\omega_i(t)t + \phi_i\right). \tag{2}$$

Here, $i = 1, 2$ denote the drive and twist pulse, respectively. The polarization of the field is $\mathbf{e}_1 = \mathbf{e}_z$ for the drive and $\mathbf{e}_2 = \mathbf{e}_x$ for the twist pulse. We describe the shape of the pulses by

$$\mathcal{E}_i(t) = \frac{\mathcal{E}_0^{(i)}}{2}\left[\tanh(a(t - t^{(i)}_{initial})) - \tanh(a(t - t^{(i)}_{final}))\right]. \tag{3}$$

For excitation schemes I and II, $\omega_1(t) = \omega_d$ and $\omega_2(t) = \omega_t$ are constant, while for scheme III a linear chirp with

$$\omega_1(t) = \omega_d - \Delta\omega + \frac{\Delta\omega}{\tau_d}t \tag{4}$$

for the drive and

$$\omega_2(t) = \omega_t - \Delta\omega - \frac{2\Delta\omega}{\tau_t}t^{(2)}_{initial} + \frac{\Delta\omega}{\tau_d}t \tag{5}$$

for the twist pulse is applied. Here, $t^{(1)}_{initial} = 0$, $\tau_d = t^{(1)}_{final}$ and $\tau_t = t^{(2)}_{final} - t^{(2)}_{initial}$ are the pulse durations for the drive and twist pulses, respectively. During the pulse, the frequency changes linearly from $\omega_{d/t} - \Delta\omega$ to $\omega_{d/t} + \Delta\omega$. Expanding the wavefunction into the eigenfunctions

of H_0,

$$|\psi(t)\rangle = \sum_n c_n(t) \exp\left(-iE_n t\right) |\varphi_n\rangle \tag{6}$$

and applying the rotating wave approximation, the Schrödinger equation reads

$$\frac{\partial}{\partial t} c_n(t) = -i \sum_m H_{nm}(t) \exp\left[-i(\Delta_{nm}^{(i)}(t)t + \phi_i)\right] c_m(t) \tag{7}$$

with $\Delta_{nm}^{(i)}(t) = E_n - E_m - \omega_i(t)$. The eigenstates of H_0, denoted by $|\varphi_n\rangle = |J_{K_a K_c}, F, M_F\rangle$ are the hyperfine states of an asymmetric top rotor, with $|J-I| \leq F \leq J+I$ and $-F \leq M_F \leq F$. For valinol with ^{14}N, the nuclear spin is $I = 1$, and we consider the hyperfine levels $1_{01}, F$, $2_{12}, F$ and $2_{02}, F$. The transition matrix elements $H_{nm} = -\sum_i \mathcal{E}_i(t) \langle\varphi_m| \boldsymbol{\mu} \cdot \mathbf{e}_i |\varphi_n\rangle$ depend on the quantum number M_F. To this end, we approximate the hyperfine level structure by an effective nine-level model where we neglect the M_F-degeneracy and consider an average transition matrix element given by

$$H_{nm} = -\sqrt{I_{nm}} \sum_i \mu_i \mathcal{E}_i(t) \tag{8}$$

with $\mu_1 = \mu_b$ and $\mu_2 = \mu_c$ and intensities I_{nm} of the hyperfine transitions as listed in.[1] In this model, the Hilbert space is spanned by the nine hyperfine states $|\varphi_n\rangle = \{|1_{01}, F\rangle\, |2_{12}, F'\rangle$, $|2_{22}, F''\rangle\}$ with $F = 0, 1, 2$ and $F', F'' = 1, 2, 3$ Note that with this approximation, we also neglect the coupling between different M_F-states occurring for excitation with x- or y-polarized fields. The validity and limitations of this model are discussed in Section 1.2. Here, the M_F-degeneracy is considered in form of the initial distribution of the hyperfine states

$$\rho_{initial} = \sum_{F=0}^{2} p_F\, |1_{01}, F\rangle \langle 1_{01}, F| \tag{9}$$

with $p_0 = 1/9$, $p_1 = 3/9$ and $p_2 = 5/9$. The signal in the frequency domain is obtained by Fourier transform of the expectation value of the induced dipole moment after the twist

pulse,

$$I(\omega) \propto \mathcal{F}\left[\langle\psi(t)|\, \boldsymbol{\mu}\cdot\mathbf{e}_y\, |\psi(t)\rangle\right] \tag{10}$$

for $t > t^{(2)}_{final}$. The Schrödinger equation (7) is numerically integrated using the program package Octave.[2] In order to match the field strength $\mathcal{E}_0^{(i)}$ to the experimental values the simulated nutations curves are compared to the experimental ones. For excitation with a single-frequency pulse, the field strength can be determined using the condition $\Theta = \mu\mathcal{E}_0 = \pi/2$ for the resonant transition. For excitation with chirped pulses, we compare the overall shape of the nutation curves to adjust the field strength - allowing a qualitative comparison between the experimental and numerical results.

1.2 Orientational degeneracy of the hyperfine states

We investigate the effect of the orientational degeneracy of the hyperfine states for a single M3WM cycle, namely $1_{01}, 2 \to 2_{12}, 3 \to 2_{02}, 3$, the level scheme including the degenerate M_F states is shown in Figure S1. The z-polarized drive pulse induces transitions with $\Delta M_F = 0$ (blue arrows). The selection rules for the x-polarized twist pulse are $\Delta M_F = \pm 1$ (red arrows). The listen signal is recorded in y- direction and the corresponding transitions are depicted with dashed green arrows. Details on simulating the microwave-driven population dynamics for degenerate states in asymmetric top rotors can be found in.[3] Initially, all degenerate M_F-states are equally populated. The z-polarized drive pulse simultaneously drives five two-level transitions with different Rabi-frequencies. This can be approximated fairly well by an average Rabi-frequency as for the nine-level model described in Section 1.1. The $x-$polarized twist pulse however, couples different degenerate M_F-states, as indicated by the red arrows in Figure S1. To describe the effect of this coupling on the listen signal, we consider a single initial state, namely $|1_{01}, 2, M_F = -2\rangle$, represented by a gray circle in Figure S1 (b) and (c). The drive pulse creates approximately a 50/50 coherence between the states $|1_{01}, 2, M_F = -2\rangle$ and $|2_{12}, 3, M_F = -2\rangle$. The twist pulse then induces $\Delta M_F = \pm 1$

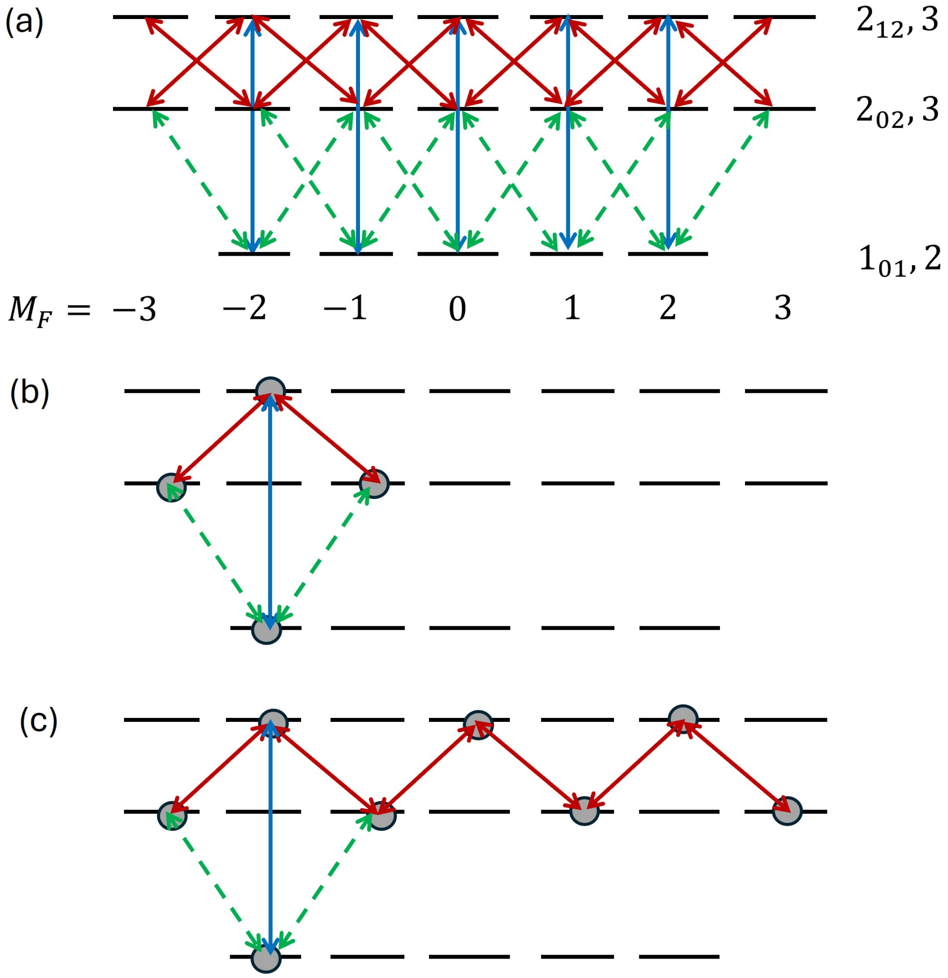

Figure S1: (a) Level scheme including degenerate M_F-states for the M3WM cycle $|1_{01}, 2\rangle \rightarrow |2_{12}, 3\rangle \rightarrow |2_{02}, 3\rangle$. The blue and red arrows indicate the transitions for drive and twist pulses. The dashed green arrows depict the listen transitions. (b) The gray circles depict the states that are populated after excitation with a drive pulse ($\tau_d = 0.3\,\mu s$) and a twist pulse ($\tau_t = 0.2\,\mu s$) for a single initial state $|1_{01}, 2, M_F = -2\rangle$. (c) Same as (b) but for $\tau_t = 1\,\mu s$.

transitions between the $2_{12}, 3$ and $2_{02}, 3$ levels. For a short twist pulse, cf. panel (b), only the neighboring states, here $|2_{02}, 3, M_F = -3\rangle$ and $|2_{02}, 3, M_F = -1\rangle$ are excited and the level scheme resembles a normal M3WM cycle. In this case, the nine-level model provides a good description of the rotational dynamics. However, for longer twist pulses, states with larger M_F will also be excited, cf. panel (c), which are not part of the M3WM cycle. Population in those states do not contribute to the listen signal. The leaking of the population to those states thus results in a decay of the listen signal for longer pulse durations, which is not covered by the effective nine-level model. The nine-level model with average transition matrix elements is thus a good description of the rotational dynamics for short twist pulses, for an adequate description of the dynamics for longer twist pulses, the complete orientational degeneracy has to be taken into account.

2 Supplementary experimental details

2.1 Pulse optimization and nutation curves

The pulse conditions were optimized by measuring nutation curves, that is, varying the pulse duration and observing the intensity of the respective hyperfine transition. In addition to the experiments, the dynamics were simulated as explained in Section 1 in more detail.

Figures S2 and S3 depict the experimentally measured and simulated nutation curves for the single-frequency pulse schemes I and II, respectively. The intensities of the different hyperfine transitions are depicted as a function of the pulse duration. The drive pulses of scheme I and II with an excitation frequency of 7874.51 MHz and 7876.08 MHz were scanned from 0 µs to 2.4 µs in steps of 0.2 µs averaging 50,000 FIDs for each data point (see panel a in Figure S2 and S3). A 3 W solid state amplifier provided the amplification power for the single-frequency drive pulses. To obtain the frequency spectrum, we performed a Fourier transformation on 30 µs of the averaged FID using a Kaiser-Bessel window ($\beta = 9.5$). The complex Fourier transform signal provides the intensity and phase of each hyperfine

component.

Due to off-resonant excitation, as discussed in the main text, not only the resonant transition can be observed, but also other hyperfine components depending on their transition matrix element and the detuning. In the experimental nutation curves, the resonant hyperfine transitions are marked with solid lines, whereas the co-excited transitions are marked with dashed lines. The drive pulses are optimized for a Rabi-flip angle of $\pi/2$, which corresponds to the maximum signal in the nutation curve for the respective transition. For both schemes, the duration of the drive pulse, τ_d was chosen such that the intensity of the resonant transition is maximal while co-excitation is minimized. Hence, we selected for scheme I a pulse duration of $\tau_{d,I} = 1.2\,\mu s$ and for scheme II, $\tau_{d,II} = 1.4\,\mu s$, as can be seen in panel a of Figure S3.

Since there is no receiving horn antenna opposite the transmitting horn antenna of the twist pulse, the durations of the twist pulses, τ_t, are optimized by measuring the listen signal using the before-determined drive durations. The twist pulses of scheme I and II were scanned from 0 µs to 3.8 µs and 0 µs to 4 µs in steps of 0.2 µs, respectively, averaging 20,000 FIDs for each data point. The measured nutation curves of the twist pulses are depicted in panel b of Figure S2 and S3. For both measured nutation curves, the hyperfine transition $F'' \rightarrow F : 2 \rightarrow 1$ could not be resolved and is therefore not present in the figure.

The maximum listen intensities correspond to a Rabi-flip angle of π of the twist pulse. A 300 W traveling wave tube amplifier set to 10% gain amplified the twist pulses. The reduced gain and therefore reduced electric field strength increases the pulse duration necessary to reach the π condition. The pulse duration is inversely proportional to the bandwidth of the pulse. Hence, longer pulse durations provide smaller bandwidths and achieve better selectivity within the narrow hyperfine-split level scheme.

Figure S4 presents the measured and simulated nutation curves for pulse scheme III. Both the drive and twist pulses were scanned in two ranges: a short scan with finer time increments and a longer scan with coarser steps. For the drive pulse (panels a and c), the

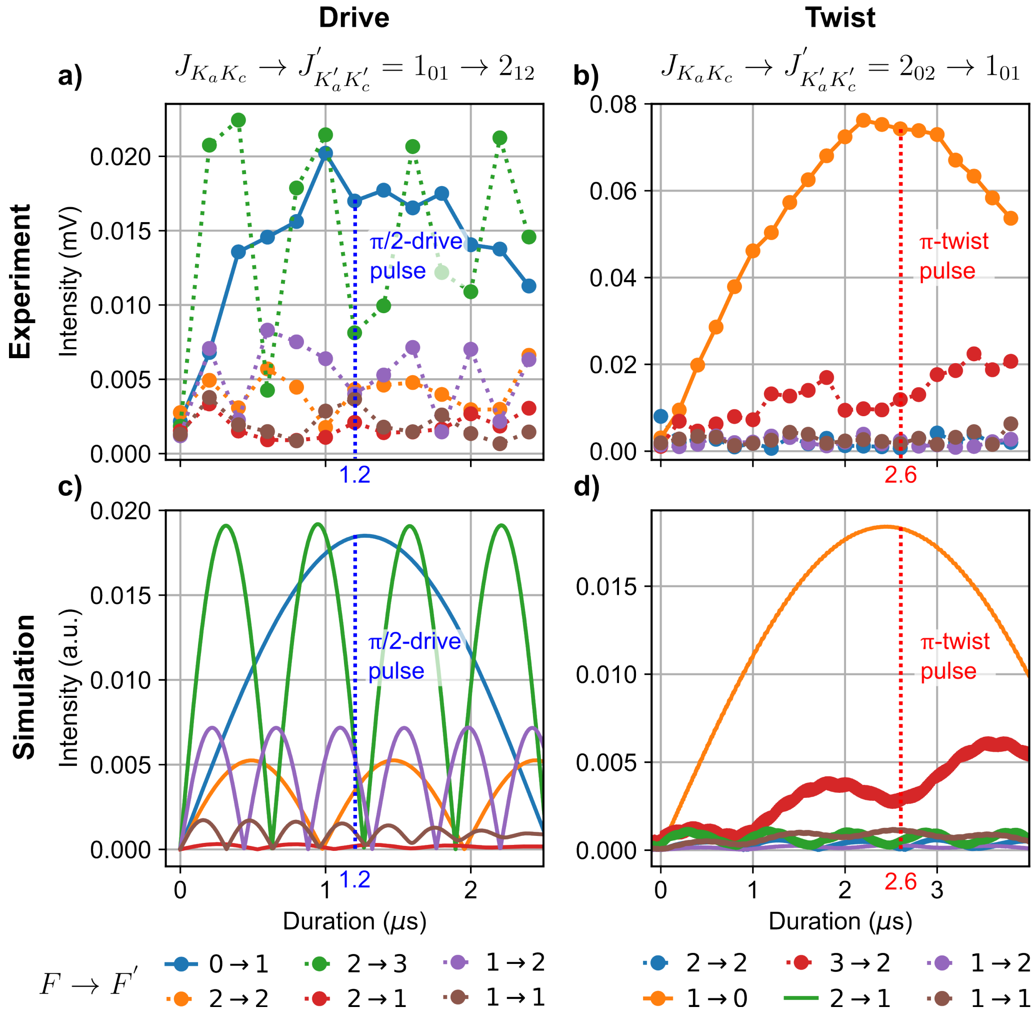

Figure S2: Experimental (top row) and simulated (bottom row) nutation curves for the drive and twist pulse for scheme I. The intensities of the different hyperfine transitions are depicted as a function of the pulse duration. In the experimental data, the signal of the hyperfine transition, which is part of the addressed M3WM cycles, is depicted with a solid line. Co-excited transitions are depicted with dashed lines.

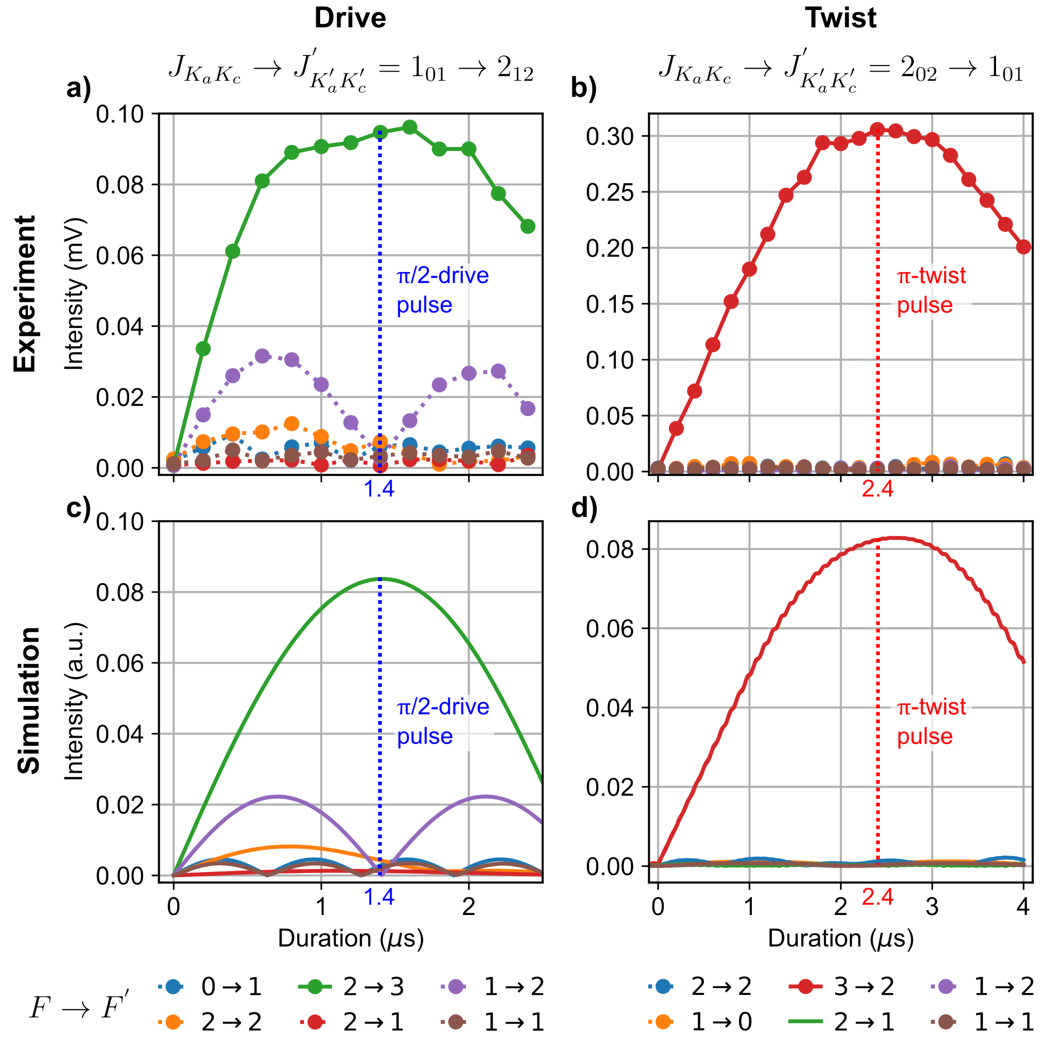

Figure S3: Experimental (top row) and simulated (bottom row) nutation curves for the drive and twist pulse for scheme II. The intensities of the different hyperfine transitions are depicted as a function of the pulse duration. In the experimental data, the signal of the hyperfine transition, which is part of the addressed M3WM cycles, is depicted with a solid line. Co-excited transitions are depicted with dashed lines.

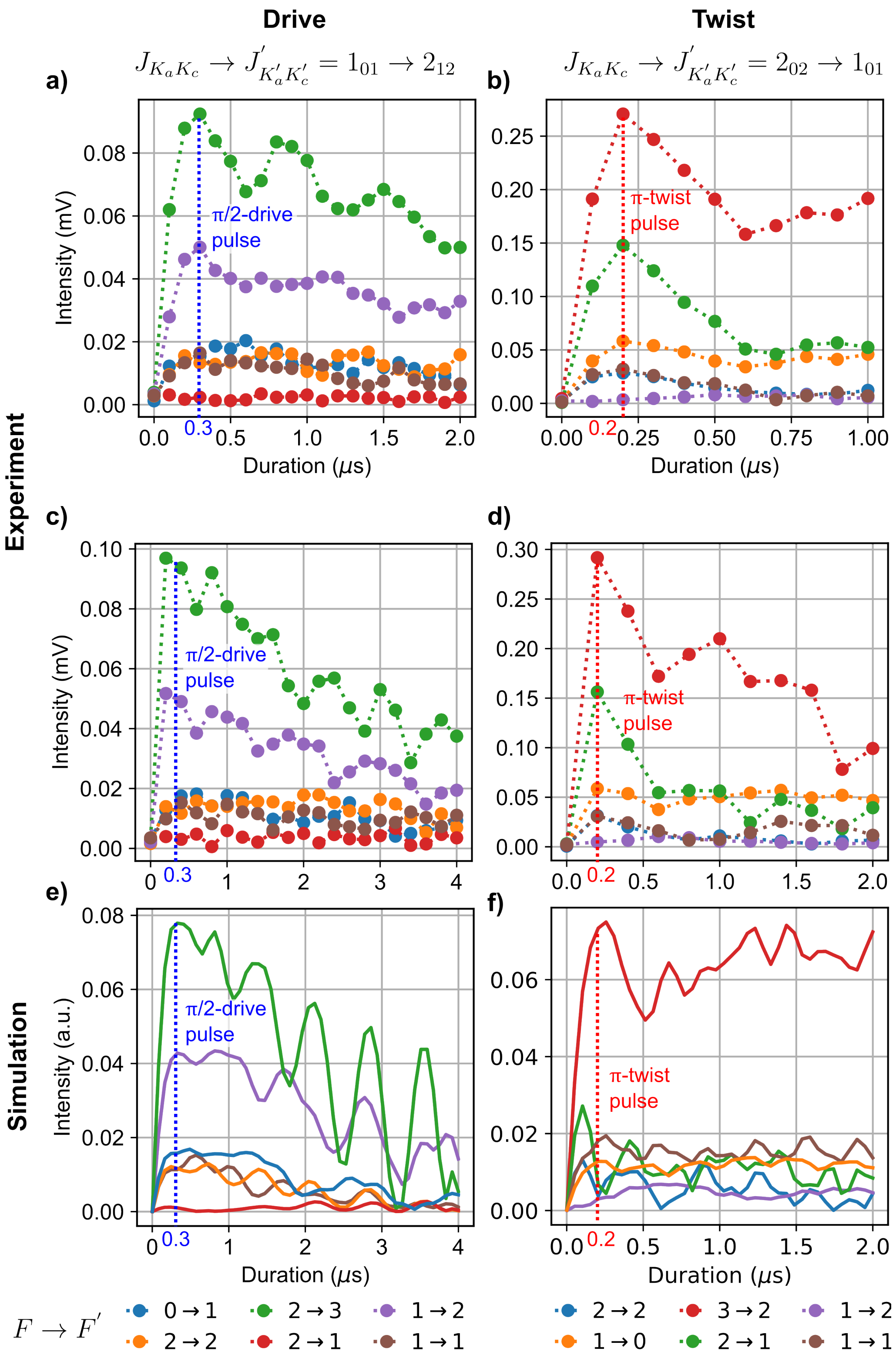

Figure S4: Experimental and simulated nutation curves for pulse scheme III. a) and c): short scan with finer step size and a long scan of the chirped drive pulse. e) simulated nutation curve of the drive pulse. b) and d) short scan with finer step size and a long scan of the chirped twist pulse. f) simulated nutation curve of the chirped twist pulse.

scan covered 0 µs to 2 µs in 0.1 µs steps (averaging 25,000 FIDs) and 0 µs to 4 µs in 0.2 µs steps (averaging 10,000 FIDs). For the twist pulse (panels b and d), the ranges were 0 µs to 1 µs in 0.1 µs steps and 0 µs to 2 µs in 0.2 µs steps, each averaging 25,000 FIDs. For both the drive and twist nutation curves, the measured intensities differ between the short and long scans, as the measurements were conducted on different days under slightly varying vacuum and pulsed molecular jet conditions. Panels e and f show the corresponding simulations based on the effective nine-level model. The measured and simulated nutation curves for the chirped drive pulse agree closely, while the twist pulse simulation does not reproduce the experimental results, as discussed in Section 1.

2.2 Phases of listen signal for scheme III

Table S1: Absolute phases ϕ_L and ϕ_D of the listen signal for L- and D-valinol and the relative phase $\Delta\phi = |\phi_D - \phi_L|$ for all hyperfine components $F'' \rightarrow F$ for scheme III.

$F'' \rightarrow F$ (GHz)	ϕ_L (deg)	ϕ_D (deg)	$\Delta\phi$ (deg)
$2 \rightarrow 2$ (5918.53)	-155	15	170
$1 \rightarrow 0$ (5918.69)	-124	55	179
$2 \rightarrow 1$ (5919.78)	-76	96	172
$3 \rightarrow 2$ (5919.84)	-24	154	178
$1 \rightarrow 2$ (5920.57)	148	-18	166
$1 \rightarrow 1$ (5921.82)	-10	167	177

Table S1 summarizes the absolute phases of each hyperfine transition of the measured listen signal for L- and D-valinol and the relative phase demonstrating enantiomer differentiation.

The graphical visualization of the molecules in the main text was performed using Chimera.[4]

References

(1) Townes, C. H.; Schawlow, A. L. *Microwave spectroscopy*; Dover Publications.

(2) Eaton, J. W.; Bateman, D.; Hauberg, S.; Wehbring, R. GNU Octave version 9.3.0 manual: a high-level interactive language for numerical computations. 2024.

(3) Leibscher, M.; Kalveram, J.; Koch, C. P. Rational Pulse Design for Enantiomer-Selective Microwave Three-Wave Mixing. *Symmetry* **2022**, *14*, 871.

(4) Pettersen, E. F.; Goddard, T. D.; Huang, C. C.; Couch, G. S.; Greenblatt, D. M.; Meng, E. C.; Ferrin, T. E. UCSF Chimera–a visualization system for exploratory research and analysis. *J. Comput. Chem.* **2004**, *25*, 1605–1612.
