# Supplementary material for: Differentiating Between Enantiomers with Nuclear Quadrupole Coupling Using Microwave Three-Wave Mixing
Source: J Phys Chem Lett. 2025 Nov 11;16(46):12087–94. doi: 10.1021/acs.jpclett.5c02849 (PMC12641466; doi:10.1021/acs.jpclett.5c02849)
Supplement: Supplementary file 2 [file jz5c02849_si_002.pdf]

Dear Prof. Jin Zhong Zhang,
Dear reviewers,

Thank you for considering our manuscript for publication in *The Journal of Physical Chemistry Letters*. We would like to thank the reviewers for their positive reports, their constructive comments and feedback that has helped us to further improve our manuscript. We have made changes accordingly (more details and a one-by-one response to the referees' comments are given below).

With kind regards,
Melanie Schnell, on behalf of all authors.

Reviewer 1

1) The simulations are performed using a reduced nine-level effective model. While this effective model aligns well with the experimental results for short pulses, the manuscript should explicitly state the parameter range within which this approximation remains reliable. A brief numerical comparison with simulations that include the full hyperfine Hamiltonian, or at least a qualitative discussion, would enhance confidence in the validity of the modeling.
→ A numerical comparison of the experiments, the nine-level model and a simulation including the M_F degeneracy is shown in Figure 5.c). We added a passage in the second paragraph on page 17, where we explicitly state the parameter range within which the nine-level model is reliable. Further, in this passage and on page 18, second paragraph, we added a qualitative discussion when an extension of the model might be necessary.

2) The manuscript presents three different pulse schemes (two single-frequency and one chirped). While the experimental results are clear, it would be beneficial for the authors to expand the comparison by discussing the practical merits and limitations of each scheme. For instance, which scheme is easier to implement experimentally? Which might scale better to more complex molecules? Which provides stronger or more reproducible signals?
→ We thank the reviewer for these suggestions and have added a passage on the advantages of the different pulse types on p. 17/18 stating that the complexity of the experimental implementation for the different pulse schemes is similar. The different pulse schemes can result in similarly strong signals, though the chirped pulses require more amplification power, which is a specific experimental demand. Chirped pulse schemes may become advantageous for even denser hyperfine structure. Single-frequency pulse schemes could be a starting point for further precision experiments that require the preparation in a specific *F* state.

3) The main claim relies on the observation of a π phase difference between enantiomers. The authors are encouraged to provide more quantitative information regarding experimental uncertainties: What is the typical error margin in phase determination (e.g., ± a few degrees)? Under conditions of lower signal-to-noise ratio, how robust is this phase signature for unambiguous enantiomeric differentiation?
→ Previous studies such as the work of W. Sun et al. in *Nat. Commun.* **14**, 934 (2023) have shown a stable behavior of the phase with a typical error margin of a few degrees even for transitions with low intensity (signal-to-noise ratio less than 10). Even if the error margin was larger, an unambiguous enantiomeric differentiation would be possible, since the overall statement relies on a difference of 180 degrees. We have added a comment in the caption of Table 3.

4) Since the study uses valinol as a benchmark system, it would strengthen the impact of the manuscript if the authors could briefly discuss the expected challenges when applying this method to heavier or more complex chiral molecules, particularly those with denser hyperfine structures or multiple quadrupolar nuclei.

→ From the numerical point of view, systems with larger nuclear spin *I* and multiple quadrupolar nuclei have a larger number of (degenerate) hyperfine states requiring simulations including the full hyperfine Hamiltonian. We have added a sentence on these challenges to the paragraph in the middle on page 17.

The density of energy levels would increase for both heavier molecules and systems with multiple quadrupolar nuclei. As already addressed in our answer to question 2), we suggest from an experimental perspective that excitation using chirped pulses would be a more straightforward approach to enantiomeric differentiation.

There is a minor typo: "loo" should be corrected to "loop" on page 9.

→ The typo has been corrected.

Reviewer 2

• *In the introduction, I find that the connection between "electronic structure" and "character of the chemical bonds" is not clearly established. The authors might emphasize this link more explicitly, rather than simply referring to Refs. 5–7.*

→ We have added more information on the link between nuclear quadrupole coupling and the electronic structure of the molecule in the introduction (page 3).

• *Is there a specific reason for choosing valinol as the demonstration case? Reference 25 reports alaninol and leucinol, which are also chiral and contain an NH2 group.*

→In general, we were aiming for a medium-sized molecule that had already been characterized using CP-FTMW spectroscopy. The choice of valinol instead of alaninol or leucinol for our experiments was based on the fact that the lowest energy conformer of valinol possesses larger dipole-moment components (μ_a, μ_b, μ_c / D = 3.0, 1.2, 0.7) compared to the lowest energy conformers of the other two molecular compounds (alaninol: μ_a, μ_b, μ_c / D = 3.1, 0.5, 0.4; leucinol: μ_a, μ_b, μ_c / D = 3.0, 0.2, 1.2).

• *Page 3: A, B, and C in the moment of inertia must be written in lowercase. The components I_a, I_b, I_c correspond to the a, b, and c principal axes, not to the A, B, and C rotational constants.*

• *Page 4: Why does the listen signal P_listen carry an operator symbol? I have the same question regarding the x-, y-, and z-polarization directions.*

• *Page 7: nuclear quadrupole coupling is abbreviated as NQC here, but the term already appears on page 3. Consider using the abbreviation consistently.*

• *Page 7: the nuclear spin I and the angular momentums J and F should be in boldface rather than written with an operator symbol [see Gordy and Cook].*

→ The four suggested corrections have been implemented.

• *This figure is somewhat difficult to interpret. The caption states that the drive transitions are highlighted in blue, twist in red, and listen in green. however, apart from the labels "Drive," "Twist," and "Listen," none of the transitions appear in these colors. This might be because the color-coding is also used to distinguish the pink and orange cycles. Is there a way to make this clear? Are the black*

dotted lines meant to indicate closed loops? In the text, the authors mention that Figure 2 illustrates both the validity and the limitations of the nine-level system. While the validity is relatively clear to me, the limitations are not.
→ We agree that the notation in the caption of Figure 2 was somewhat confusing and have adjusted it by removing the term "highlighted". The black dotted lines depict all dipole allowed transitions, in general (this detail has been added to the caption), while the orange and pink colors mark closed cycles that are further discussed in the text.
It is the energy scheme that the nine-level model is based on, that is depicted Figure 2, not its validity and limitation – we have rephrased the sentence for more clarity and added a discussion on the limitations of the model on page 17.

• *The authors attribute the weaker listen signal of D-valinol relative to L-valinol to "a lower degree of enantiomeric purity of the commercial sample." What is the enantiomeric purity specified by Santa Cruz Biotechnology and Sigma-Aldrich?*
→ Both companies have been contacted but they could not provide any details on the enantiomeric purity but only the overall chemical purity (99.9% for *L*-valinol, 98.5% for *D*-valinol). However, it has been observed by several researchers working with chiral molecules that commercial samples of chiral molecules, even though denoted as *D*-valinol or *L*-valinol, can be not enantiopure, but rather enantioenriched. In general, a calibration of the listen signal can solve the problem of signal variations. However, as for this work only the phases are relevant such a calibration was not performed. Since it is a speculation, based on experience, that the sample might not be enantiopure, we rephrased the sentence on page 12 to a more general statement.

• *Page 14: The manuscript states that the chirped pulses "excite all the possible listen transitions," but shouldn't they instead excite all possible drive and twist transitions, with the listen transitions being detected rather than excited?*
→ The reviewer is right, the sentence has been corrected accordingly.

• *Until the end, it remains unclear to me, which scheme should be the best. If one were to repeat the same experiment on another molecule with 14N hyperfine structure, which scheme would the authors recommend?*
→ We have added a passage on the advantages of the different pulse types on p. 17/18, see also our reply to the 2nd comment of reviewer 1.

• *Typos: page 6: drive, twist, and listen transition -> drive, twist, and listen transitions; page 8: the nine-level system presented in Figure 2 shows the its validity -> delete "its" or "the"; page 10: closes the loo -> closes the loop; Figure 4 caption: state -> states*
→ All the typos have been corrected.
